# Strategies to Improve the Efficiency of Transplantation with Mesenchymal Stem Cells for the Treatment of Ischemic Stroke: A Review of Recent Progress

**DOI:** 10.1155/2021/9929128

**Published:** 2021-08-27

**Authors:** Yongchang Li, Wei Zhong, Xiangqi Tang

**Affiliations:** Department of Neurology, The Second Xiangya Hospital, Central South University, Changsha, Hunan 410011, China

## Abstract

Cerebral ischemia is a common global disease that is characterized by a loss of neurological function and a poor prognosis in many patients. However, only a limited number of treatments are available for this condition at present. Given that the efficacies of these treatments tend to be poor, cerebral ischemia can create a significant burden on patients, families, and society. Mesenchymal stem cell (MSC) transplantation treatment has shown significant potential in animal models of ischemic stroke; however, the specific mechanisms underlying this effect have yet to be elucidated. Furthermore, clinical trials have yet to yield promising results. Consequently, there is an urgent need to identify new methods to improve the efficiency of MSC transplantation as an optimal treatment for ischemic stroke. In this review, we provide an overview of recent scientific reports concerning novel strategies that promote MSC transplantation as an effective therapeutic approach, including physical approaches, chemical agents, traditional Chinese medicines and extracts, and genetic modification. Our analyses showed that two key factors need to be considered if we are to improve the efficacy of MSC transplantation treatments: survival ability and homing ability. We also highlight the importance of other significant mechanisms, including the enhanced activation of MSCs to promote neurogenesis and angiogenesis, and the regulation of permeability in the blood-brain barrier. Further in-depth investigations of the specific mechanisms underlying MSC transplantation treatment will help us to identify effective methods that improve the efficiency of MSC transplantation for ischemic stroke. The development of safer and more effective methods will facilitate the application of MSC transplantation as a promising adjuvant therapy for the treatment of poststroke brain damage.

## 1. Introduction

Ischemic stroke is a major global disease that is associated with high incidence and mortality rates. The most common cause of ischemic stroke is a blockage of the cerebral vessels; this often leads to disability, thus placing a huge socioeconomic burden on patients, families, and society [1]. Thrombolytic therapy with recombinant tissue plasminogen activator (rt-PA) is the only treatment approved by the United States-food and drug administration (FDA) for acute ischemic stroke; however, this drug is not widely used clinically due to its restricted time window of efficacy (4.5 hours) and unpredictable complications [2, 3]. Therefore, there is an urgent need to identify novel treatment options to address the limitations associated with current treatment strategies for ischemic stroke [4, 5].

Cell transplantation treatments have shown great potential in animal models to improve the sequelae of neurological diseases. In particular, stem cell transplantation therapy has become a major area of research over recent years. Studies have found that stem cells are immortal and can proliferate into neurons and glial cells when used to treat diseases of the central nervous system, thereby replacing necrotic cells and exerting neuroprotective effects [6]. Mesenchymal stem cells (MSCs) have become the most widely used form of stem cells in biological medical research, largely because of their neuroprotective effects on ischemic stroke in animal models [7–9].

MSCs are multipotent adult stem cells that can self-renew and can be found in a variety of tissues, including bone marrow, adipose tissue, and the umbilical cord [10, 11]. The ease with which MSCs can be isolated from a diverse range of tissues is one of the main advantages of MSC-based therapies. Compared with other stem cell-based therapies, MSCs have several unique advantages. MSCs possess the ability to migrate to an area of damage and can be transplanted before differentiating into mature cells [12, 13]. Furthermore, MSCs with “plastic” immune properties (immunostimulatory and immunosuppressive) can be used in a wider range of applications [14]. Given their ability to cross allogeneic barriers, MSCs may become an “off-the-shelf” stem cell that could be used in certain emergency clinical situations [14]. Furthermore, a considerable body of evidence now supports the fact that MSCs can improve recovery by enhancing angiogenesis and neurogenesis and by inducing immunomodulatory and anti-inflammatory effects [15]. In addition, the paracrine actions of MSCs are known to promote functional recovery *via* both direct and indirect effects [15, 16].

However, stem cell therapy for transplantation is associated with poor efficacy. Recent studies have shown that after the transplantation of stem cells, only a small number of stem cells reach the damaged area; furthermore, these cells tend to disappear quickly. This indicates that the efficacy of this method is compromised by the poor survival rates of the donor cells [17–19]. In this review, we summarize research progress in in the neuroprotective approaches that can be used to improve the efficiency of MSC transplantation for ischemic stroke.

### 1.1. The Immunomodulatory Effects of MSCs

Inflammatory signaling is involved in all stages of cerebral ischemia, from the early damaging events triggered by vascular occlusion to the late regenerative processes underlying postischemic tissue repair [20]. Ischemic stroke induces a strong inflammatory response, prompting a large number of leukocytes to accumulate in the ischemic infarct area [20]. In contrast to other types of stem cells, MSCs possess the capability to mediate the immune response. When MSCs enter an inflamed brain, they can be become anti-inflammatory cells by reducing the secretion of tumor necrosis factor alpha (TNF-*α*) and by increasing the secretion of interleukin-10 (IL-10) [21]. The transplantation of MSCs was found to reduce infarct size and improve functional deficits in a mouse model of middle cerebral artery occlusion (MCAO) by immunomodulating the expression of the IL-23/IL-17 axis [22]. Moreover, the transplantation of MSCs has also been shown to suppress inflammatory responses (by reducing the levels of IL-1*β*, IL-6, and TNF-*α*) and neuronal apoptosis during the early stages of focal cerebral ischemia in rabbits [23]. Li et al. confirmed that MSCs restrain astroglial activity in the peri-ischemic area and increase the expression levels of IL-10 to inhibit ischemic injury and improve neurological function [24]. The implantation of MSCs into the injured brain enhances neuroprotection by activating the activity of NF-kappaB (NF-*κ*B) in resident stem cells, thus leading to an increase in IL-6 production and a decrease in apoptosis [25]. In addition to regulating the expression of cytokines to induce immunosuppression, MSCs can also modulate the immune response by inhibiting B cells [26, 27]. Moreover, inflammatory cell (T cell) proliferation was found to be reduced after coculture with MSCs *in vitro*, thus suggesting that the nitric oxide (NO) produced by MSCs is one of the major mediators of T cell suppression [28].

### 1.2. The Secretion of Paracrine Factors by MSCs

MSCs have the potential to differentiate into adipocytes, osteoblasts, chondrocytes, and neurons [29]. Furthermore, transplanted MSCs have been shown to migrate to the infarct zone and differentiate into neuronal, glial, and endothelial cells to enhance neuroplasticity [30]. Moreover, the paracrine action of MSCs has been shown to induce regenerative processes by increasing the level of growth factors or receptors [31]. MSC treatment also increases the expression of stromal cell-derived factor-1 (SDF-1), vascular endothelial growth factor (VEGF), brain-derived neurotrophic factor (BDNF), and growth-associated protein-43 (GAP-43), in the peri-infarct region [32].

Furthermore, intravenously transplanted MSCs are known to induce functional improvement and reduce infarct volume in ischemic rats, possibly by providing insulin-like growth factor 1 (IGF-1), inducing vascular endothelial growth factor (VEGF), epidermal growth factor (EGF), and basic fibroblast growth factor (bFGF), neurotrophic factors in the host brain [33]. IGF-1 plays an important role in neurological recovery by promoting neurogenesis; MSC transplantation has also been shown to increase the expression levels of IGF-1 and IGF-1 receptors in ischemic brain tissue [33, 34]. These bioactive factors promote functional recovery after stroke in synergistic manner. In addition, the administration of MSCs increases endogenous brain bone morphogenetic protein 2/4 and connexin-43 expression in astrocytes and promotes the expression of synaptophysin; these factors facilitate functional recovery in rats following stroke [35]. Collectively, these data may indicate that the endocrine effects of MSCs may mitigate difficulties in sending sufficient numbers of MSCs to the ischemic infarct area. This may suggest that the endocrine effects of MSCs mitigate the typical difficulties faced in enabling sufficient numbers of MSCs to reach the ischemic infarct area.

## 2. Physical Approaches to Improve the Efficiency of MSC Transplantation

Oxygen is an essential element for cellular homeostasis; hypoxia has been shown to be involved in the pathological processes of many diseases associated with high morbidity and mortality rates [36]. Bone marrow, one of the most common sources of MSCs, is a hypoxic environment with an oxygen tension of approximately 1% to 7% [37]. Hypoxic pretreatment is a simpler and easier approach than other methods such as genetic modification. Many studies have found that moderate hypoxia preconditioning can improve the cellular activities of MSCs [38]. In a rat model of ischemic stroke, hypoxic preconditioning of bone marrow MSCs (BMSCs) improved their ability to promote cell homing, neuronal differentiation and regeneration, and the recovery of neuronal function *via* CXCL12/CXC chemokine receptor type 4 (CXCR4) signaling [39–41]. Moreover, experiments involving hypoxic-preconditioned BMSCs in a rat model of MCAO demonstrated the upregulation of hypoxia-inducible factor-1*α* (HIF-1*α*) and a number of trophic/growth factors (e.g., BDNF, VEGF, and glial cell-derived neurotrophic factor [GDNF]) and the downregulation of proinflammatory cytokines/chemokines [40]. Further research confirmed that ischemic rat cortical neurons could be rescued by coculturing them with hypoxic-preconditioned BMSCs [42]. In addition, hypoxic preconditioning also reduced the extent of apoptosis in BMSCs during ischemia–reperfusion (I/R) injury by inhibiting caspase-3 activation and increasing the expression of HIF-1*α* [41]. In a rat model of cardiac arrest-induced global cerebral ischemia, hypoxia was shown to promote the migration and integration of MSCs, rescued a greater number of neurons, and suppressed inflammation in the ischemic brain by activating the phosphoinositide-3 kinase (PI3K)/AKT and HIF-1*α*/CXCR4 pathways [43]. Furthermore, granulocyte colony-stimulating factor (G-CSF) improved the efficiency of hypoxic preconditioning in promoting the survival and migration of canine BMSCs, thus indicating that a combination of the two interventions (G-CSF and hypoxic preconditioning) may offer a novel strategy for improving the efficiency of MSC transplantation [44]. Collectively, this evidence indicates that hypoxic preconditioning improves the homing ability (*via* CXCL12/CXCR4 signaling) and survival ability (antiapoptotic and anti-inflammatory properties) of transplanted MSCs, thus helping MSCs to fulfill their therapeutic function in a more efficient manner.

Other physical strategies that improve the homing ability of MSCs have also been reported. Ultrasound-targeted microbubble destruction can disrupt biological barriers and increase vascular permeability, including the blood-brain barrier (BBB) [45]; this technique may help more transplanted cells to migrate to the injured brain area. Qian et al. found that ultrasound-targeted microbubbles enhanced the homing effect of BMSCs during the application of transplantation to treat ischemic stroke [46]. This improved level of homing led to the amelioration of cerebral edema, a reduction in infarct size, and an improved neurological function score in a rat model of MCAO. Moreover, the application of ultrasound microvesicles to assist MSC infusion facilitated the homing of BMSCs to the cerebral infarct and reduced the infarct volume by upregulating VEGF expression and by modulating apoptosis [47].

**Figure 1:**
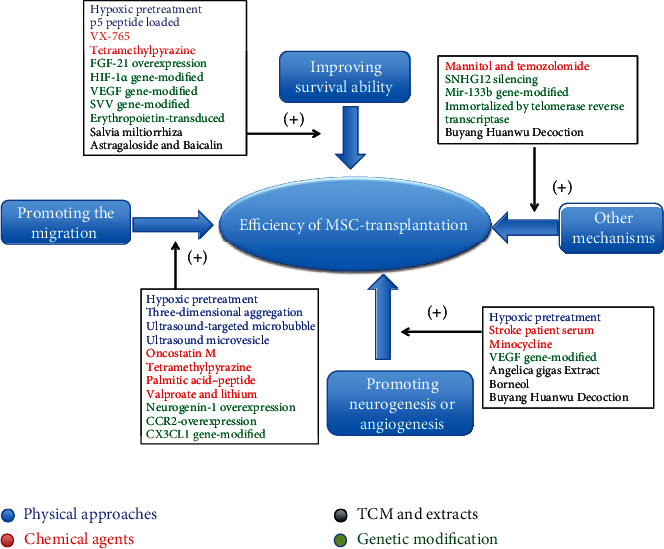
Possible main mechanisms of strategies to improve efficiency of MSC transplantation in ischemic stroke models.

Improving MSCs so that they can adapt and survive better in the harsh environment of the ischemic brain would increase the efficiency of MSC transplantation treatment. A previous study showed that the short-term preconditioning of human mesenchymal stem cells (hMSCs) *via* three-dimensional (3D) aggregation promoted resistance to oxidative stress while also restoring energy homeostasis and innate cellular properties [48]. These effects were partly mediated *via* regulation of the PI3K/AKT pathway and demonstrated that preconditioning improves the therapeutic efficacy of hMSCs in the treatment of ischemic stroke. In another study, Paudyal et al. reported that p5, a 24-residue peptide derived from p35, offers protection to neurons and endothelial cells *in vitro* [49]. These authors administered human adipose-derived mesenchymal stem cell- (hADMSC-) loaded p5 peptide in a rat model of focal cerebral ischemia; they found that this promoted the survival of transplanted hADMSCs and enhanced neurological recovery. The main mechanisms of action underlying the physical approaches used to improve the efficiency of MSC transplantation are summarized in Table 1 and Figure 1.

## 3. The Use of Chemical Agents to Improve the Efficiency of MSC Transplantation

The use of chemical agents to increase the transplantation efficiency of MSCs has also become a significant research topic. Tetramethylpyrazine (TMP) is a biologically active alkaloid that is extracted from the rhizome of the Chinese herb Rhizoma Chuanxiong [50]. Owing to its antioxidant, antiapoptotic, and anti-inflammatory properties, TMP has become a widely accepted therapeutic agent for cardiovascular and cerebrovascular diseases in China [50, 51]. In a series of *in vitro* experiments, TMP was shown to improve the survival of BMSCs against H_2_O_2_-induced apoptosis by regulating the PI3K/AKT and extracellular regulated protein kinases1/2 (ERK1/2) signaling pathways [52]. Furthermore, in a rat model of ischemic stroke, BMSCs combined with TMP promoted the homing of BMSCs towards ischemic areas by upregulating the expression of SDF-1 and CXCR4. In addition, this combined treatment increased the secretion of nerve growth factors (BDNF and VEGF) and promoted angiogenesis and neurogenesis, thus leading to improved functional recovery after cerebral ischemia [53].

Increased transplantation treatment efficiency requires more transplanted cells to survive and function in a harsh environment. Previous studies have shown that VX-765, a novel small molecule caspase-1 inhibitor, is able to suppress inflammation by passing the BBB [54]. In addition, this drug has been used clinically in a phase IIa randomized, double-blind, multicenter, placebo-controlled trial to treat epilepsy [55]. Furthermore, an in-depth study by Sun et al. demonstrated that the pretreatment of human umbilical cord mesenchymal stem cells (HUMSCs) with VX-765 improved cell survival during transplantation treatment by upregulating autophagy *via* the AMP-activated protein kinase (AMPK) and mammalian target of rapamycin (mTOR) signaling pathway, thus ameliorating inflammatory responses, inhibiting apoptosis, and reducing infarct volume in HUMSC-transplanted models of stroke [56]. These researchers suggested that pretreatment with VX-765 may become a novel method for improving the efficacy of MSC-based regenerative therapies for cerebral ischemia. Minocycline, a second-generation tetracycline, is known to exert a neuroprotective effect on ischemic stroke by inhibiting the activity of matrix metalloproteinase [57]. Recent experiments confirmed that a therapeutic treatment that combined human bone marrow-derived MSCs (hBMSCs) and minocycline promoted neurological recovery and reduced infarct volume, possibly by upregulating neuronal nuclear antigen (NeuN) and VEGF at the boundary of the ischemic area [58]. These results suggested that minocycline may become an adjuvant therapy for improving the efficiency of transplantation treatment.

Improving the ability of MSCs to migrate is still the key approach for improving their transplantation efficiency. The suppression of matrix metalloproteinase-9 (MMP-9) was reported to attenuate the ability of stem cells to migrate to the infarcted brain [59]. In contrast, priming with valproate and/or lithium was shown to improve the migration ability of MSCs by activating CXCR4 and MMP-9 [60]. In turn, this improved the efficiency of transplantation treatment by reducing infarct volume, enhancing angiogenesis, and by improving functional recovery in a rat model of cerebral ischemia. Both valproate and lithium have been used to treat bipolar disorder [61, 62]; additional supporting evidence will help to further confirm the importance of these factors to MSC transplantation treatment for ischemic stroke. Oncostatin M (OSM), a member of the IL-6 cytokine family [63], has been shown to bind to specific receptor complexes and activate particular signaling pathways to regulate downstream events [63]. In addition, OSM preconditioning has been shown to improve MSC migration, possibly by enhancing the secretion of growth factors and cytokines [64, 65]. In a recent study, a combined treatment featuring OSM and BMSCs was shown to upregulate SDF-1 to improve BMSC migration and neurofunctional recovery in a rat model of ischemic stroke by promoting the expression of VEGF and MMP-2 and by reducing the expression of inflammatory mediators [66]. In addition, researchers have shown that painting cell membranes with palmitic acid-peptide enhanced the targeted migration of MSCs to ischemic brain tissue and reduced the accumulation of cells in peripheral organs, thus leading to improved therapeutic efficiency [67]. These results indicate that modification of the cell surface with targeting peptides may offer a novel approach to increase the efficacy of MSCs in ameliorating ischemic brain injury.

Adjusting the permeability of the BBB is a novel method to help MSCs reach injured areas of brain tissue. Using a rat model of MCAO, researchers have shown that the combination of mannitol and temozolomide improved the efficacy of human umbilical cord-derived MSCs (hUC-MSCs) in the treatment of chronic cerebral ischemia and ameliorated behavioral deficits, possibly by improving neural regeneration and angiogenesis [68]. The principal mechanism underlying the effects induced by this combined drug treatment is likely to involve increased BBB permeability. However, the increased level of permeability in the BBB that results from ischemic damage during acute ischemic stroke is commonly considered to be the primary cause of hemorrhagic transformation and is associated with a worse outcome [69, 70]. Therefore, increasing BBB permeability could be a controversial way of overcoming obstacles to the clinical application of MSCs in patients with acute ischemic stroke.

Activating MSCs to promote neurogenesis and angiogenesis more effectively is another novel approach that could be deployed to improve the efficiency of transplantation treatment. Bone marrow stromal cells that are isolated from rat models of stroke are superior to those from normal rats when used for the transplantation treatment of cerebral ischemia; this may be due to the enhanced expression of trophic factors and the increased angiogenic characteristics of BMSCs from the rat model of stroke [71]. These findings suggest that serum obtained from the rat model of stroke activates allogeneic MSCs. Moon et al. demonstrated that culture methods for MSCs using serum acquired from stroke patients were able to improve the behavioral outcome in a rat model of stoke stroke by promoting neurogenesis and angiogenesis [72]. These effects may have been mediated by the increased expression levels of VEGF, glial cell-derived neurotrophic factor (GCNF), and fibroblast growth factor (FGF), in MSCs that were cultured with serum from stroke patients. Furthermore, another study found that transplanting a plasma-derived scaffold combined with BMSCs into the cystic cavity led to significantly reduced atrophy volume and improved motor function in a rat model of MCAO, as compared with rats treated with a vehicle, the scaffold, or BMSCs only [73]. These results suggest that components of the blood (such as the plasma-derived scaffold and serum from stroke patients) may offer a promising means to improve the efficiency of MSC transplantation in the treatment of cerebral infarction. The main mechanisms of action for the chemical agents described herein are summarized in Table 2 and Figure 1.

## 4. Traditional Chinese Medicine and Extracts for Improving the Efficiency of MSC Transplantation

Traditional Chinese medicine (TCM) has been widely used in China for treating a variety of diseases. TCM preparations (Chinese materia medica) and their active compounds have been shown to ameliorate brain damage induced by ischemia [74]. Over recent years, several studies have investigated the beneficial effects of TCM with regard to the efficiency of MSC transplantation treatment for cerebral infarction.

In addition to playing a protective role in ischemic stroke, Buyang Huanwu decoction (BHD) has also been shown to protect the BBB by inactivating glycogen synthase kinase 3 (GSK-3) and Tau [75, 76]. The combination of BHD and MSC transplantation was also shown to be more effective at treating injured blood vessels and ischemic tissues and acted *via* the upregulation of VEGF and Ki-67 expression [77]. Furthermore, BHD is known to augment angio-miRNA in MSC-secreted exosomes *in vitro* and promote angiogenesis, as observed in a rat model of ischemic stroke [78]. Collectively, these results suggest that the combination of BHD and MSCs could represent a useful adjuvant therapy for ischemic stroke.

Salvia miltiorrhiza (SM) is a popular plant that is widely used in TCM for the treatment of various diseases. SM was recently shown to ameliorate ischemic brain damage, possibly *via* multiple processes including antiplatelet aggregation, and both anti-inflammatory and antioxidative effects [79]. In a study involving the treatment of cerebral infarction with MSC transplantation, SM was shown to promote the antiapoptotic ability and survival of MSCs, improve transplantation efficiency, and significantly improve recovery from ischemic stroke [80]. Astragaloside, the main active substance of Astragalus, has also been shown to improve the antiapoptotic and anti-inflammatory abilities of MSCs under inflammatory conditions when combined with baicalin [81]. Furthermore, the combination of astragaloside IV and tanshinone IIA was shown to improve the migration of MSCs and promote the homing of MSCs *in vitro*, possibly *via* the modulation of CXCR4 expression [82].

In addition, the combination of borneol and MSC transplantation has been shown to effectively suppress apoptosis, reduce infarct volume, and promote neurogenesis, thus providing a neuroprotective effect during functional recovery in a rat model of MCAO [83]. Angelica gigas (AG) is a widely used herbal medicine; the extract of AG has been shown to exert a neuroprotective effect on ischemic stroke-related injury in the brain [84]. In a rat model of MCAO, Kim et al. found that AG extract and MSCs act synergistically to increase the overall therapeutic effect by enhancing neovascularization [85].

Collectively, these results suggest that the addition of TCM enhances the efficiency of MSC transplantation treatment by enhancing the homing ability or survival of the transplanted MSCs. However, the putative ability of TCM to improve the therapeutic efficacy of MSCs still needs to be supported by further evidence. Although these TCM treatments still require more *in vivo* data to confirm their efficacy, they are expected to become novel and effective methods for improving the efficiency of MSC transplantation in the treatment of ischemic stroke. The main mechanisms of action related to these TCM approaches are summarized in Table 3 and Figure 1.

## 5. Genetic Modification Approaches to Improve the Efficiency of MSC Transplantation

Improving the homing ability of MSCs is an important key goal of gene editing for transplantation treatment. Neural-induced MSCs have been shown to be more effective than parental MSCs with regard to neurological recovery in a rat model of MCAO [86]. Moreover, neural induction *via* a retroviral vector expressing the neurogenic transcription factor neurogenin-1 (Ngn1) was shown to increase the homing ability of MSCs and improve their engraftment efficiency in a rat model of MCAO [87]. These results suggest that the neural induction of MSCs by genetic modification may provide a novel method for improving the efficiency of MSC transplantation. Fractalkine (FKN), also known as CX3CL1, is a chemokine that is constitutively expressed in the brain [88]. Zhang et al. found that the FKN–CX3CR1 chemokine axis could activate Jak2–Stat5–ERK1/2 signaling and improve the directional migration of MSCs towards ischemic cerebral lesions [89]. These results indicate that the combination of gene-modified MSCs with chemokines could provide a novel approach to improve the efficiency of MSC transplantation treatment. Recent experimental evidence now supports the fact that the overexpression of CCR2 can enhance the targeted migration of MSCs to ischemic brain tissue and improve their therapeutic efficiency by reducing ischemic lesions and improving neurological recovery [90]. These effects have been attributed to alleviated inflammatory infiltration and PRDX4-mediated antioxidant effects that mediate BBB preservation.

Activating MSCs to resist harsh environments more effectively and to exhibit enhanced levels of functionality is the key aim of genetically modifying MSCs. Hypoxia-inducible factor 1 (HIF-1) is an important transcription factor that plays a key role in the cellular response to hypoxic environments [91]. HIF-1*α*, a subunit of HIF-1, modulates several target genes related to angiogenesis, energy metabolism, apoptosis, autophagy, and other adaptive responses to hypoxia [92, 93]. The transplantation of BMSCs stably expressing mutant HIF-1*α* (P564A and N803A) was found to efficiently promote functional recovery and reduce infarct volume in a rat model of MCAO, possibly by increasing the expression levels of VEGF [94]. Therefore, the mutation of HIF-1*α* may represent a novel target for improving the treatment of stroke with MSC transplantation. Moreover, Lv et al. used MSCs that had been modified with the HIF-1*α* gene and reported that the overexpression of HIF-1*α* protects MSCs against oxygen-glucose deprivation- (OGD-) induced injury by promoting cell viability, suppressing apoptosis, and by activating autophagy-related signaling pathways (PI3K/AKT/mTOR); these effects were reversed by knocking down HIF-1*α* [95, 96]. Further experiments, using a rat model of MCAO, showed that the overexpression of HIF-1*α* in MSCs reduced infarct volume and improved the neurobehavioral outcome when compared with control MSCs; these effects occurred by the inhibition of proinflammatory cytokines and the promotion of autophagy and neurotrophin secretion [95].

FGF-21, a metabolic regulator involved in the regulation of gluconeogenesis and lipid metabolism, has been shown to protect cells from the excitotoxicity induced by ischemic stroke [97, 98]. Linares et al. found that the overexpression of FGF-21 improved the survival of transplanted MSCs and reduced apoptosis under the harsh microenvironment associated with oxidative stress [99]. However, more experimental data are required to confirm that FGF-21 improves the efficiency of stroke treatment involving the transplantation of MSCs. Survivin (SVV), an inhibitor of apoptosis, has been shown to play a protective role in many cellular metabolic activities [100, 101]. Furthermore, SVV-engineered MSCs were found to not only improve cardiac performance in a rat model of myocardial infarction [102], but also promoted the survival of MSCs during transplantation treatment for stroke [103]. These authors also found that, when compared with parental MSCs, SVV gene-modified MSCs improved the functional recovery of a rat model of MCAO and reduced cerebral infarct volume. *In vitro* experiments further suggested that these effects were mediated by the increased secretion of protective cytokines (VEGF and bFGF) from the gene-modified MSCs [103]. Erythropoietin (EPO) is a type of glycoprotein hormone that stimulates and promotes the production of red blood cells. EPO may exert a neuroprotective effect on ischemic stroke and protect the BBB from ischemic damage by increasing the expression of p-Connexin43 [104, 105]. Further experiments have demonstrated that MSCs transfected with the EPO gene are able to continuously secrete EPO and various neurotrophic factors, thus improving the viability of neural cells and transplanted MSCs [106]. Furthermore, the implantation of EPO-MSCs was found to improve neurological deficits and reduce infarct volume in a rat model of MCAO [106].

In addition, approaches to editing telomerase and RNA have also become significant research topics in the application of MSC transplantation. As the telomeres in hMSCs gradually become shorter during the process of continuous replication, the ability of hMSCs to self-renew is limited; this may affect the stability of the genome and the metabolic function of the hMSCs [107]. Li et al. attempted to immortalize hMSCs using a human telomerase reverse transcriptase and found that immortalized cells performed better during the treatment of transient cerebral ischemia than control hMSCs; the immortalized hMSCs reduced apoptosis in brain cells, reduced infarct volume and brain edema, and improved neurological recovery [108]. MicroRNAs (miRNAs) are a form of single-stranded and noncoding RNA that exert a crucial regulatory effect on molecular processes following ischemic stroke [109, 110]. Moreover, a large number of miRNAs have been shown to regulate the migration of MSCs in animal models of a variety of diseases; these effects occur *via* a range of mediatory mechanisms including cytokine–receptor interaction, cytoskeleton remodeling, intracellular signaling, and MSC-derived exosomes [111]. The treatment of a rat model of MCAO with miR-133b-positive MSCs was shown to improve functional recovery when compared with rats that received treatment with naive MSCs, while also promoting axonal plasticity and neurite remodeling in the ischemic boundary zone; these effects were reversed by the administration of miR-133b-negative MSCs [112]. The mechanism underlying these effects may relate to the mediation of miR-133b transfer to astrocytes and neurons *via* exosomes from the MSCs. Long noncoding RNAs (lncRNAs), a class of noncoding mRNA transcripts that are >200 nucleotides in length and do not encode proteins [113], have also become a significant focus of research with regard to the use of gene editing approaches to improve the efficiency of MSC transplantation for the treatment of stroke. Li et al. conducted a series of experiments using lncRNAs and found that the silencing of SNHG12 in MSCs promoted cell proliferation and reduced apoptosis in cocultured brain microvascular endothelial cells; these effects occurred *via* the activation of the PI3K/AKT/mTOR signaling pathway [114]. Furthermore, compared with control MSCs, SNHG12-silenced MSCs reduced the infarct volume and significantly reduced apoptosis in a rat model of MCAO [114]. The main mechanisms of action involved in these genetic modification approaches are summarized in Table 4 and Figure 1.

## 6. Clinical Trials of MSC Therapy for Ischemic Stroke

First application of autologous BMSCs transplantation in patients with ischemic stroke is reported in 2005 [115]. In this study, the Barthel index and modified Rankin score of the MSC group (5 patients) improved consistently during the follow-up period and no adverse reactions were reported [115]. In 2010, Lee et al. reported that intravenous injection of BMSCs resulted in better recovery and reduced mortality for up to 5 years from treatment initiation, compared with randomized controls [116]. Another study evaluated efficacy of administration of serum-expanded autologous BMSCs to chronic stroke patients and got similar results in 2011 [117]. Furthermore, Steinberg et al. conducted a phase I/II study of intracerebral cell transplantation in patients with chronic stroke and reported that intracerebral transplantation of genetically modified MSCs significantly improved neurological function [118, 119], whereas Jaillard et al. did not report an overall benefit in a single-center, open-label randomized controlled trial study [120]. In 2019, Levy et al. completed a separate phase I/II trial in which patients received intravenous injection of allogeneic single-donor BMSCs [121]. Excellent functional outcome was reported in 35.5% of patients at 12-month postinfusion, compared to in 11.4% at baseline; however, there was no control group included in this study. Although MSC administration has been shown to be safe and feasible in small, early phase trials, questions about the efficacy of MSC treatment for ischemic stroke remain [122]. No significant improvement was observed in a randomized controlled intravenous phase II trial [122]. In 2021, Chung et al. reported that MSC treatment is not associated with improvements in the 3-month modified Rankin score, and their study provided class III evidence that autologous MSCs do not improve 90-day outcomes in patients with chronic stroke [123].

In summary, these studies support the safety of MSCs for transplantation in patients with ischemic stroke, but the therapeutic efficacy remains controversial. More optimized and well-designed large sample multicenter studies are needed to determine the effect of MSC therapy in the treatment of ischemic stroke. The identification of optimal transplantation protocol for routine clinical applications, including the route of administration, the concentration of cells, the timing, patient selection criteria, and combination therapies, will help us to further identify effective and safe therapeutic methods for ischemic stroke.

## 7. Limitations and Future Directions

Although MSC transplantation has demonstrated good prospects for the treatment of cerebral ischemia, there are still clear limitations with regard to the treatment of ischemic stroke. First, the administration of BMSCs primarily involves intracranial transplantation *via* stereotactic delivery, intravenous injection, or intra-arterial injection. Intracranial transplantation is an invasive treatment that can cause additional damage to the ischemic brain; in particular, infarct areas often require multiple injections. Although intravascular injection is a minimally invasive treatment option, this type of transplantation prevents many MSCs from reaching the area of cerebral ischemia, as most of the transplanted cells tend to accumulate in the peripheral organs [124, 125]. Second, the potential tumorigenic risks of BMSC transplantation require attention and further investigation [126, 127]. Third, there are also limitations related to the sources of MSCs that are currently available. Cerebral infarction often occurs in elderly patients; it is difficult to obtain a sufficient number of healthy autologous MSCs from elderly patients or from patients with a range of severe diseases [128]. Fourth, whether the transplanted MSCs can successfully differentiate into fully functional neurons has yet to be determined [129]. Finally, the limited replicative lifespan of MSCs also limits the efficacy of transplantation therapy [130]. Therefore, if we are to improve the efficacy of MSC transplantation, a series of targeted studies will need to be carried out. For example, it is important that we fully evaluate methods that could enhance the homing efficiency and survival rate of MSCs. In addition, future studies should also focus on improving the probability of successful differentiation and expanding the replicative lifespan of MSCs.

During preclinical studies, the transplantation of MSCs has demonstrated beneficial effects in several neurological and motor skills tests. However, the encouraging data derived from animal models cannot necessarily be translated into clinical practice [131]. Studies involving preclinical experiments are usually performed by applying standardized protocols for lesions in each study group. These conditions cannot be fully replicated in human patients suffering from ischemic stroke. Most strokes that occur in humans, particularly those that are treatable, are significantly smaller in size than those observed in preclinical models [132, 133]. Different types of donor cells may have also contributed to the differential results arising from preclinical and clinical studies. For example, most studies involving animal models used fresh MSCs that were isolated from healthy and young donors. However, many of the clinical studies used autologous MSCs instead of those derived from healthy individuals [131]. MSCs derived from healthy donors have been demonstrated to be more efficacious than those isolated from nonhealthy donors [134, 135].

In addition, future research should focus on the timing of MSC transplantation; this may constitute a significant challenge with regard to translating basic science studies into clinical practice. Most studies involving preclinical experiments have focused on the acute administration of MSCs. However, all the patients involved in clinical studies were administered with MSC therapy during the subacute or chronic phase following stroke [131]. Moreover, patients who have experienced cerebral infarction often have comorbidities such as hypertension and diabetes. Consequently, these patients need to take antidiabetic medicine and antiplatelet drugs; these drugs could influence the functionality of the MSCs, thus limiting their therapeutic effects [136]. Finally, senescence may also exert influence upon the therapeutic effects of MSCs; this is related to the limited number of passages used in the isolation of these cells. Extending the time available for extension will inevitably lead to replicative senescence [130]. Therefore, future studies should also focus on developing strategies that can address senescence in MSCs.

## 8. Conclusion

Currently, targeted treatments for cerebral infarction are associated with limited efficacy. As such, there is a clear need to identify novel treatment methods. MSC transplantation has demonstrated significant potential in animal models of MCAO, although efficacy still needs to be improved further. In this article, we reviewed recent progress in research studies aiming to improve the efficacy of MSC transplantation to treat the poststroke brain. A number of approaches have been proposed, including physical methods, chemical agents, traditional Chinese medicines and extracts, and genetic modifications. The latest research evidence has identified two key factors that must be considered if we are to improve efficacy: the survival and homing ability of MSCs after transplantation. Moreover, activating MSCs to promote neurogenesis and angiogenesis and adjusting the permeability of the BBB have also been found to be effective treatment methods. The identification of novel methodology and gaining a deeper understanding of the specific mechanisms underlying improved MSC transplantation treatments will help us to further identify effective and safe therapeutic methods for cerebral ischemia.

## Figures and Tables

**Table 1:** Recent studies reporting physical approaches that can improve the efficiency of MSC transplantation in ischemic stroke models.

Physical approaches	Possible mechanisms	Animal model	Source of MSC	Research time	References
Hypoxic pretreatment	Promoting cell homing by CXCL12/CXCR4 signaling pathway.	Rat cerebral infarction model	Bone marrow (rat)	2019	Hu et al. [39]
Hypoxic pretreatment	Promoting the migration and integration of MSCs by PI3K/AKT and HIF-1*α*/CXCR4 pathways	Rat cardiac arrest-induced global cerebral ischemia model	Bone marrow (rat)	2017	Wang et al. [43]
Hypoxic pretreatment	Improving survival and inhibiting apoptosis by inhibiting of caspase-3 activation and increasing expression of HIF-1*α*	Rat cerebral infarction model	Bone marrow (rat)	2017	Chen et al. [41]
Hypoxia/reoxygenation pretreatment	Promoting cell migration and increasing their ability to rescue ischemic cortical neurons.	Cell model of cerebral ischemia	Bone marrow	2015	Kim et al. [42]
Hypoxic pretreatment	Promoting the expression of trophic/growth factors and improving regenerative responses.	Rat cerebral infarction model	Bone marrow (rat)	2012	Wei et al. [40]
p5 peptide loaded	Promoting survival of transplanted MSCs	Rat cerebral infarction model	Adipose (human)	2020	Paudyal et al. [49]
Three-dimensional aggregation	Promoting the migration and resistance to oxidative stress by PI3K/AKT pathway	Rat cerebral infarction model	Bone marrow (human)	2019	Yuan et al. [48]
Ultrasound-targeted microbubble	Promoting the migration of MSCs	Rat cerebral infarction model	Bone marrow (rat)	2019	Qian et al. [46]
Ultrasound microvesicle	Promoting the migration of MSCs	Rat cerebral infarction model	Bone marrow (rat)	2017	Chang et al. [47]

**Table 2:** Recent studies reporting chemical agents that can improve the efficiency of MSC transplantation in ischemic stroke models.

Chemical agents	Possible mechanisms	Animal model	Source of MSC	Research time	References
VX-765	Improving the cell survival by upregulating of autophagy via AMPK/mTOR	Rat cerebral infarction model	Umbilical cord (human)	2020	Sun et al. [56]
Oncostatin M	Promoting migration ability of MSCs by upregulating SDF-1	Rat cerebral infarction model	Bone marrow (rat)	2019	Han et al. [66]
Tetramethylpyrazine	Promoting cell homing by CXCL12/CXCR4 signaling pathway.	Rat cerebral infarction model	Bone marrow (rat)	2019	Li et al. [53]
Minocycline	Upregulating NeuN and VEGF in ischemic area boundary	Rat cerebral infarction model	Bone marrow (human)	2018	Cho et al. [58]
Mannitol and temozolomide	Increasing the BBB permeability	Rat cerebral infarction model	Umbilical cord (human)	2018	Choi et al. [68]
Stroke patient serum	Promoting neurogenesis and angiogenesis	Rat cerebral infarction model	Bone marrow (human)	2018	Moon et al. [72]
Palmitic acid-peptide	Promoting migration ability of MSCs	Rat cerebral infarction model	Bone marrow (rat)	2017	Huang et al. [67]
Tetramethylpyrazine	Promoting survival of MSCs against H_2_O_2_-induced apoptosis by PI3K/AKT and ERK1/2 pathways	Cell model of cerebral ischemia	Bone marrow (rat)	2017	Fang et al. [52]
Valproate and lithium	Promoting cell homing and migration ability by activating CXCL12/CXCR4 and MMP9	Rat cerebral infarction model	Not mentioned	2011	Tsai et al. [60]

**Table 3:** Recent studies reporting TCM and extracts that can improve the efficiency of MSC transplantation in ischemic stroke models.

Drug name	Extraction source	Possible mechanisms	Animal model	Source of MSC	Research time	References
Salvia miltiorrhiza	Salvia miltiorrhiza Bunge	Promoting the antiapoptotic ability and survival of MSCs	Rat cerebral infarction model	Bone marrow (rat)	2018	Kim et al. [80]
Angelica gigas extract	Angelica gigas	Enhancing neovascularization	Rat cerebral infarction model	Bone marrow (rat)	2018	Kim et al. [85]
Borneol	Artemisia and Dipterocarpaceae	Suppressing apoptosis and promoting neurogenesis	Mouse cerebral infarction model	Fetal mice	2017	Zhang et al. [83]
Astragaloside and baicalin		Improving the antiapoptosis and anti-inflammatory ability of MSCs by MAPK/ERK pathway	Cell inflammatory model induced by lipopolysaccharide	Bone marrow (mice)	2017	Zhu et al. [81]
Buyang Huanwu decoction	TCM prescription	Augmenting angio-miRNA in MSC-secreted exosomes	Rat cerebral infarction model	Not mentioned	2015	Yang et al. [78]
Buyang Huanwu decoction	TCM prescription	Upregulating VEGF and Ki-67	Rat cerebral infarction model	Bone marrow (rat)	2010	Zhang et al. [77]

**Table 4:** Recent studies reporting genetic modification approaches that can improve the efficiency of MSC transplantation in ischemic stroke models.

Genetic modification approaches	Possible mechanisms	Animal model	Source of MSC	Research time	References
FGF-21 overexpression	Improving the survival of MSCs and reducing apoptosis	Cell oxidative stress and inflammation model	Bone marrow (mice)	2020	Linares et al. [99]
Neurogenin-1 overexpression	Increasing the homing ability and engraftment efficiency of MSCs	Rat cerebral infarction model	Bone marrow (human)	2020	Kim et al. [87]
Immortalized by human telomerase reverse transcriptase	Reducing apoptosis in brain	Rat cerebral infarction model	Bone marrow (human)	2019	Li et al. [108]
SNHG12 silencing	Promoting the cell proliferation and reduce apoptosis of brain microvascular endothelial cells	Rat cerebral infarction model	Bone marrow	2019	Li et al. [114]
CCR2-overexpression	Promoting migration ability of MSCs	Rat cerebral infarction model	Bone marrow (human)	2018	Huang et al. [90]
HIF-1*α* gene-modified	Promoting viability, suppressing apoptosis, and activating autophagy-related signaling pathway	Rat cerebral infarction model and cell OGD model	Bone marrow (rat)	2017	Lv et al. [95, 96]
VEGF gene-modified	Improving the survival of MSCs, increasing expression and secretion of VEGF and BDNF	Rat cerebral infarction model	Bone marrow	2017	Zong et al. [137]
CX3CL1 gene-modified	Promoting migration ability of MSCs	Rat cerebral infarction model	Bone marrow (rat)	2015	Zhang et al. [89]
MiR-133b gene-modified	Exosomes from MSCs mediating the miR-133b transfer to astrocytes and neurons	Rat cerebral infarction model	Bone marrow (rat)	2013	Xin et al. [112]
SVV gene-modified	Improving the survival of MSCs and upregulating the expression of protective cytokines	Rat cerebral infarction model	Bone marrow (rat)	2011	Liu et al. [103]
Erythropoietin-transduced	Improving the survival of MSCs	Rat cerebral infarction model	Bone marrow (human)	2010	Cho et al. [106]

## Additional Points

*Highlights*. (1) Existing literature was reviewed to identify strategies to improve the efficiency of MSC transplantation for ischemic stroke. (2) We summarize the specific mechanisms underlying the efficacy of strategies to improve the efficiency of MSC transplantation. (3) Improving the survival and homing abilities of MSC posttransplantation is vital if we are to improve treatment efficacy.
